# Book Reviews

**Published:** 1894-01

**Authors:** 


### *BOOK REVIEWS.*

---

A TEXT-BOOK OF PHYSIOLOGY. By M. Foster, M. D., F. R. S. Professor of Physiology in the University of Cambridge, and Fellow of Trinity College, Cambridge. Fifth edition, thoroughly revised. Lea Bros. & Co., Philadelphia.

Foster's Physiology has long been a standard work. Previous editions have been carefully reviewed in these pages. For the present edition many changes and additions have been made without affecting the character of the original work. These changes and additions are not intended so much to add new matter or enlarge the general scope of the work as to explain more fully and at greater length the most important and fundamental topics. The author nowhere pays too much attention to histological detail, but confines himself very wisely to the *physiology* of the structures described.

Foster's Physiology has for many years enjoyed and deserved a widespread popularity, and it gives us pleasure to recommend it to all in need of a standard treatise on this subject.

